# Beetle gloss is a cue of conspecifics, but only in sunny conditions 

**DOI:** 10.1098/rsbl.2026.0358

**Published:** 2026-10-21

**Authors:** Amanda M. Franklin, Aika H. Young, Katrina J. Rankin, Aëto Apaix, Mark Hanlon, Avichal Tatu, Casper J. van der Kooi

**Affiliations:** ^1^ Department of Ecological, Plant and Animal Sciences, La Trobe University, Melbourne, Australia; ^2^ School of BioSciences, The University of Melbourne, Parkville, Australia; ^3^ College of Nursing and Health Sciences, Flinders University, Adelaide, Australia; ^4^ Ecole Normale Supérieure de Lyon, Lyon, France; ^5^ Department of Collections and Research, Western Australian Museum, Welshpool, Australia; ^6^ Groningen Institute for Evolutionary Life Sciences, University of Groningen, Groningen, The Netherlands

**Keywords:** specular reflectance, glossiness, weather, illumination, communication, flash

## Abstract

Illumination conditions have a tremendous influence on the appearance of colours in nature. This is especially true for glossy objects, which generate bright highlights in direct sunlight, but the visibility of these highlights is noticeably reduced in shaded or overcast conditions. Despite this dramatic change in appearance with illumination, we know little about how illumination influences the visibility of glossy objects, and no study has directly tested the functional consequences. Here, we investigated whether jewel beetles (family: Buprestidae) use glossiness as a long-distance cue of conspecifics and how weather conditions determine the visibility of glossy beetles. We documented beetle attraction to glossy and matte interception traps in their natural habitat. Male beetles were more attracted to glossy than matte traps, but only on sunny days. To determine whether surface gloss is also used to locate conspecifics at short-range, we documented beetle behaviour towards glossy and matte replica beetle models placed on host trees. Beetles were more likely to approach glossy than matte replica beetles. Our results indicate that jewel beetles use glossiness as both long- and short-range cues of potential conspecifics, but only in sunny conditions. This demonstrates that environmental conditions determine when these brilliant visual cues are functionally relevant.

## 1. Introduction

Gloss is ubiquitous in the natural world, from shiny beetles and butterflies to brilliant mammalian fur and glossy flowers, leaves and fruits [1–8]. Glossy effects are created by flat and smooth surfaces that reflect a significant proportion of incoming light at the mirror angle (i.e. an angle equal and opposite to the angle of incidence) [1,9]. A high degree of surface gloss can be used by animals as a cue of a resource, independent from other visual cues such as object colour [10–13]. For example, bees can use flower gloss to locate potential food sources [10] and robber flies can use the frequency of flashes off an insect's wing during flight to identify suitable prey [13]. Surface gloss can be particularly effective as a long-distance cue because glossy objects reflect a very high proportion of light within a narrow angular range [1,9]. As a result, the bright reflections can contrast strongly with the surrounding environment and should be detectable from greater distances than matte objects [14]. However, despite several studies demonstrating that gloss can be an effective cue, none have empirically investigated the influence of illumination conditions on function.

The conspicuousness of glossy objects strongly depends on the illumination [15], which can vary with time of day and weather conditions. For a glossy object, if the illumination comes from a specific angle (e.g. directional sunlight), most light will be reflected at an equal but opposite angle, producing a bright flash of light (figure 1). However, if incoming light is coming from a wider field (e.g. on an overcast day), light will be reflected across a wider angular space, causing a spatially consistent, matte appearance (figure 1). In other words, in direct sunlight the visibility of glossy reflections will be maximized, whereas during overcast weather or in the shade surface gloss is hardly visible. Surface gloss thus probably functions as a resource cue only in direct sunlight.

**Figure 1.**
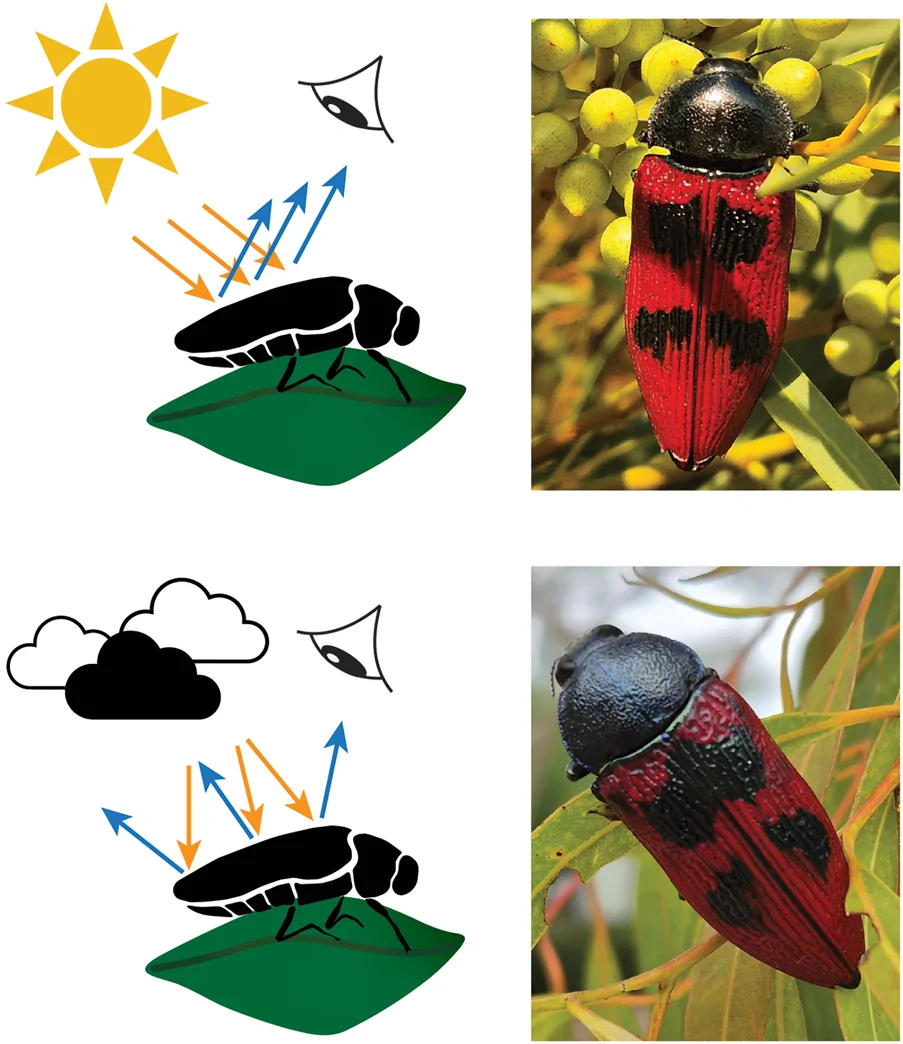
A glossy appearance is prominent in direct sunlight and hardly appreciable in cloudy conditions. Glossy objects reflect a large portion of incoming light at the mirror angle (i.e. an angle equal and opposite to the angle of incidence; blue arrows). In sunny conditions, when illumination is directional (i.e. orange arrows), light is reflected at the same angle, creating a glossy appearance. Under cloudy conditions, illumination is wide-field and reflected light is spatially homogeneous, rendering surface gloss much less conspicuous.

Here, we quantified long- and short-distance attraction of glossy *Temognatha bonvouloirii* (family: Buprestidae; Saunders [16]) jewel beetles to glossy or matte stimuli under natural field conditions. We created blue interception traps and replica beetles that mimicked the spectral and spatial reflectance characteristics of blue *Temognatha* beetles (supplementary material, figure S1). Interception traps were used to investigate the impact of gloss on long-distance attraction under sunny and cloudy conditions (figure 2). Replica beetles were used to determine the impacts of glossiness on short-distance attraction and reproductive behaviour (figure 3). Our results show how the type of weather shapes the efficacy of visual cues and signals.

**Figure 2.**
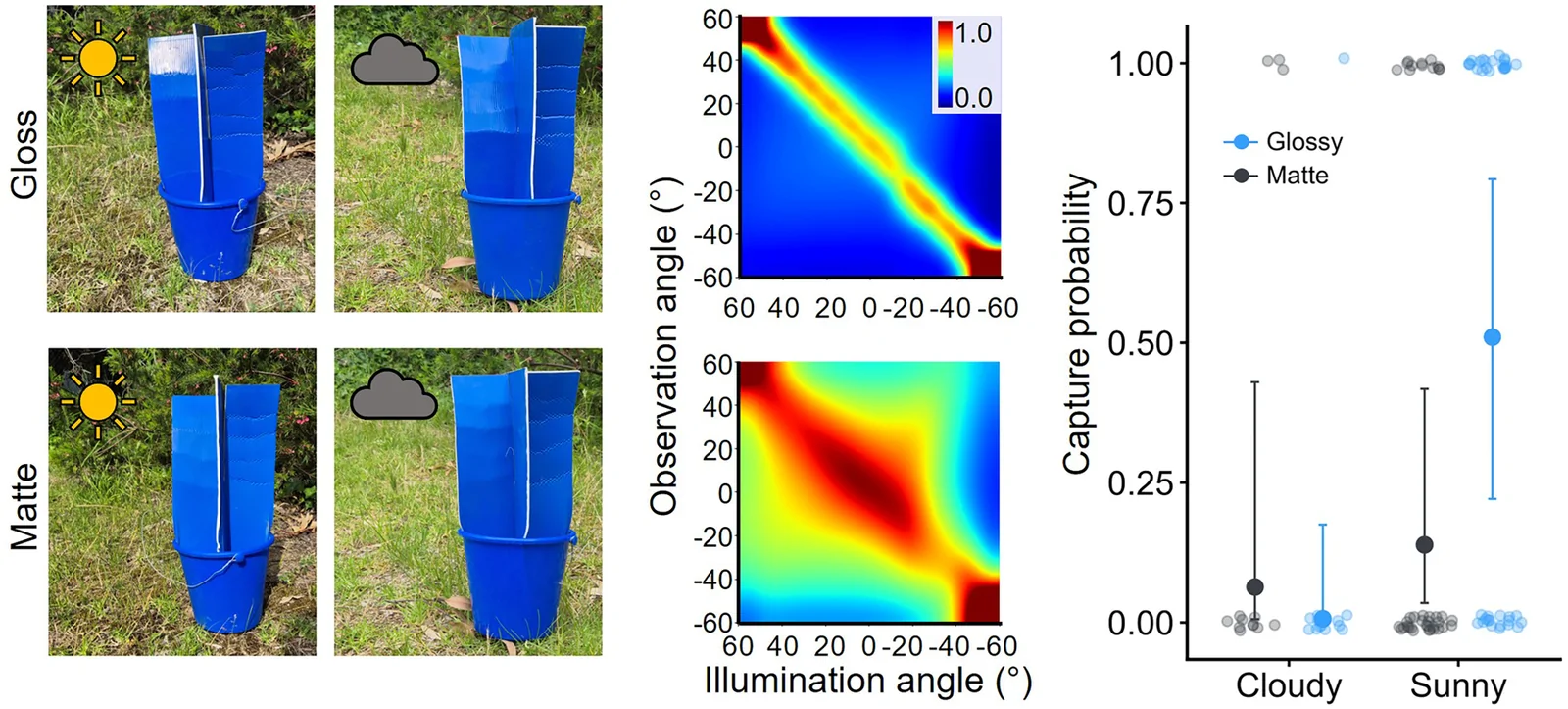
Experiment 1, long-distance attraction: beetles were more attracted to glossy than matte traps, but only in sunny conditions. Glossy and matte traps photographed in sunny and cloudy conditions. Note that the top of the front-facing insert is curved to increase the range of directions from which gloss is detectable. Heat maps show that glossy traps reflect most light at the specular angle (indicated by the dark red diagonal; dark red indicates maximum reflectance), whereas for matte traps reflectance is more spatially heterogeneous. Graphs depict maximum-likelihood estimates and 95% confidence intervals, and transparent points show raw data.

**Figure 3.**
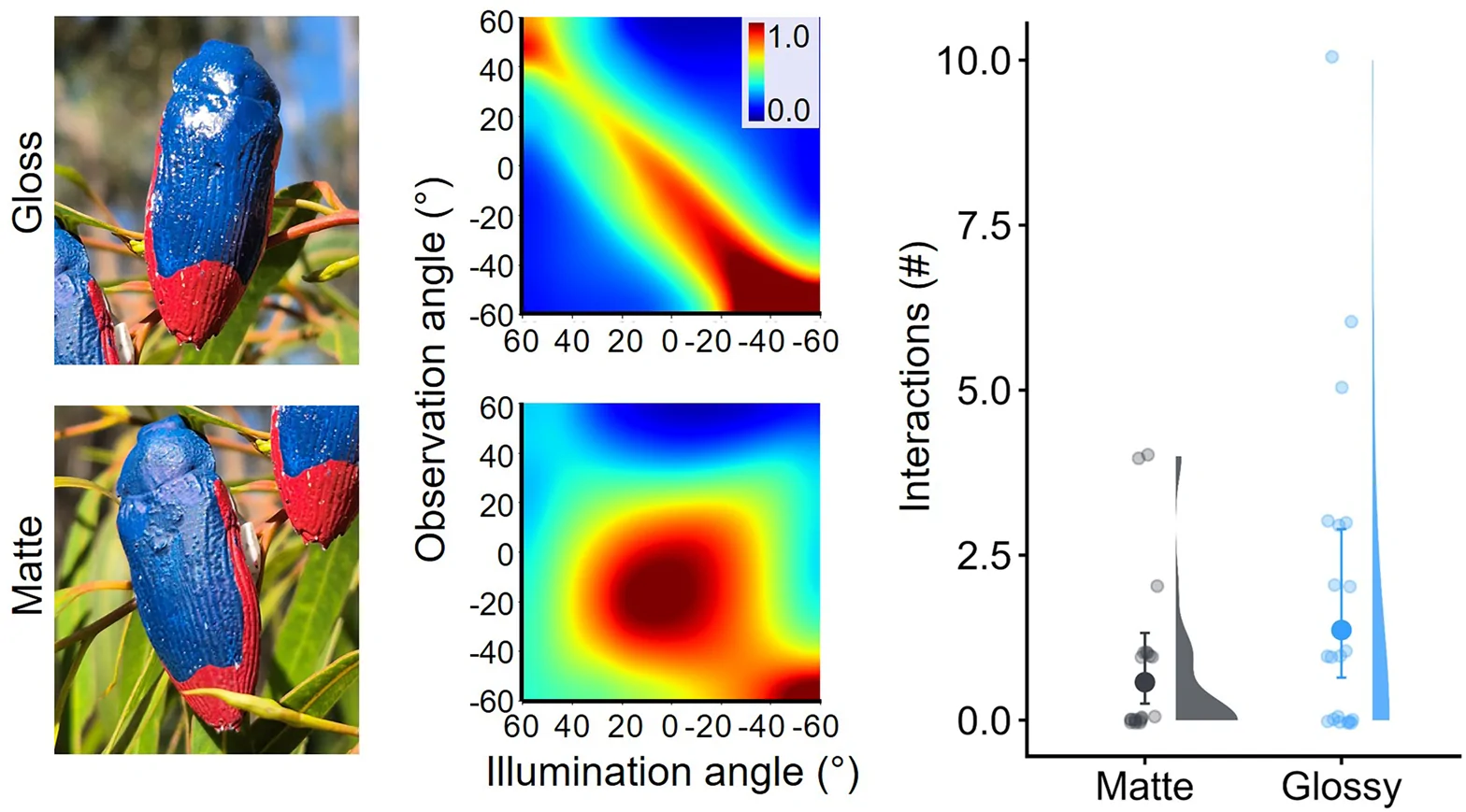
Experiment 2, short-distance attraction: beetles were more attracted to glossy replicas. Glossy and matte replica beetles photographed in sunny conditions. Heat maps show that glossy replicas reflect most light at the specular angle (indicated by the dark red diagonal; dark red indicates maximum reflectance), whereas for matte replicas reflectance is more spatially heterogeneous. Graphs depict maximum-likelihood estimates and 95% confidence intervals, and transparent points show raw data.

## 2. Material and methods

### (a) Field site

*Temognatha* buprestid beetles are often found on flowering *Eucalyptus* trees (e.g. *E. leptopoda*, *E. rigidula* and *E. cylindriflora*) in semi-arid regions during summer in Australia. The location, abundance and species of beetle present vary year to year, as do the species of *Eucalyptus* in flower. In late January 2025, we located large numbers of *Temognatha* near Wubin in the Wheatbelt region of Western Australia (30°2′0.78″ S, 117°10′14.22″ E). We established our field site along a 10 km stretch of road by visually locating *Eucalyptus* trees with *Temognatha* beetles on the flowers.

### (b) Reflectance measurements

Several *Temognatha* species are known to be attracted to traps of a specific colour, usually similar to their own body colour (AMF & MH 2000–2024, personal observations). Therefore, for both experiments, we designed stimuli that were similar in blue to real buprestid beetles. For experiment 1, chromatic characteristics of the traps were similar to three blue jewel beetle species that are known to be attracted to blue: *T. bonvouloirii, T. mniszechi* and *T. conspicillata.* For experiment 2, we colour-matched our replica beetles to *T. bonvouloirii* because this species is often abundant at our field sites.

To create stimuli of colour and glossiness similar to real beetles, we measured spectral and spatial reflectance characteristics of stimuli and beetles, and compared spectral shape and spread of reflected light. As for many beetles (reviewed in [7]), *Temognatha* beetles create blue via structural coloration, which—unlike pigment-based colour—is preserved in dried specimens. Therefore, reflectance properties of real beetles were recorded from specimens collected during our 2024 fieldwork (measurement details in supplementary material). For experiment 1, we selected a blue vinyl (Oracal 551 marine blue PVC film, Orafol, NSW, Australia) that was most similar in colour to the three target species, and for experiment 2, we selected a mix of acrylic paints that produced blue and red colours similar in spectral shape to *T. bonvouloirii* (supplementary material). To create glossy and matte treatments, the traps or replica models were sprayed with clear glossy or matte finish varnish. A series of angle-dependent reflectance measurements (see supplementary material for details) was used to quantify the spatial reflectance characteristics (i.e. ‘glossiness’) [17]. Reflectance of glossy traps and replicas was highest at the mirror angle (heat maps in figures 2 and 3; reflectance is highest at the mirror angle, i.e. the observation angle is equal and opposite to the illumination angle), corresponding to a bright flash of light at this angle. Matte traps and replicas reflected light across a wider angular space (heat maps in figures 2 and 3), resulting in a matte appearance.

### (c) Experiment 1: long-distance attraction

To investigate whether gloss influences long-distance attraction of *Temognatha* beetles, we quantified attraction to glossy and matte interception traps. Interception traps were created from blue plastic buckets (Big W, Australia) with two corrugated plastic inserts (Corflute, Bunnings, Australia) covered in blue vinyl and slotted together to create an X (figure 2). The top of one insert was curved to increase the range of directions that the gloss would be visible from. All buckets had a small amount of water in the bottom (approx. 4 cm) to ensure that beetles could not escape after they entered the trap.

At each location with suitable host trees, we set up two or four interception traps in the sun and within 30 m of a host tree. Four traps (i.e. two pairs of traps) were set up if there were multiple host trees with at least 15 active beetles. If there were fewer host trees, only two traps were set up (i.e. one pair of traps). For each glossy and matte trap pair, we identified two positions at a similar distance to the host tree with similar environmental characteristics. Traps were preferentially hung from branches of dead trees because this ensured that the buckets were at a similar height to active beetles, visible and in the sun. If this was not possible, we hung traps from branches of live trees, ensuring traps were in the sun. At some sites, trees were all quite small (less than 3 m) and bushy. At these sites, we hammered three stakes into the ground and taped the bucket traps to these stakes (approx. 1.2 m high). Within each pair of traps, both the glossy and matte traps were always erected using the same method (i.e. both on dead trees, both on live trees or both on stakes). We collected trap data for eight consecutive days, and during this time 16 trap pairs were established.

Traps remained in position for 48 h; however, sampling periods only occurred in the morning because beetles were inactive after late morning, probably owing to extreme heat (more than 40°C). Traps needed to be checked roughly every 2 h to avoid beetles drowning in the water. Therefore, every morning there were two sampling periods: early morning (traps established between 07.00 and 08.20) and late morning (traps established between 08.40 and 11.30). Before the first sampling period and during each check of the traps, we removed any insects in the traps, replaced evaporated water, and ensured the inserts were in place. Once the traps were set, they remained in place for 2.5 h, on average. At this time, all *Temognatha* beetles were counted, sexed and collected to avoid double trapping (beetles were released at the conclusion of the experiment), and then trap treatments were switched with the paired position for the second sampling period. At each check, we also recorded weather conditions (i.e. sunny, partly sunny and cloudy). This protocol was repeated the following day, with the opposite combination of treatment, position and sampling period. Therefore, there were four sampling periods for each trap position.

Before analysis, we removed unreliable collection periods (see supplementary material) and extracted records only from male *T. bonvouloirii*, because catch numbers for the other focal species were low owing to low abundance. We then ran two generalized linear mixed models (GLMMs) in R v. 4.4.0 [18]. The first investigated whether trap gloss (glossy, matte), weather conditions (sunny or cloudy) or an interaction between these factors influenced trap success (i.e. presence or absence of male *T. bonvouloirii*; binomial error distribution). The second model investigated whether trap gloss influenced the number of beetles in each trap (count data; Poisson error distribution) and was conducted only for the traps that captured at least one beetle. The latter model did not include data from cloudy days because few traps captured beetles on cloudy days (*n* = 4); therefore, only trap gloss was included as a predictor variable. We included sampling duration (85–250 min, 145 min average) as a covariate in the binomial GLMM and an offset in the Poisson GLMM. We also included pair ID (i.e. a value indicating traps in paired positions) and position ID (i.e. a value indicating position within pair) as random effects, but position ID was removed for the binomial model to avoid singularity. Diagnostic plots were used to assess model fit. Significance of fixed effects was assessed using Wald's chi-squared tests, and differences among groups were evaluated using means and 95% confidence intervals [19,20].

### (d) Experiment 2: short-range attraction

We investigated the impact of glossy reflections on short-range attraction by observing beetle interactions with replica beetle models. Replica beetle models were created from real beetles following our previously established protocol (supplementary material) [21]. Briefly, we created moulds of four *T. bonvouloirii* beetles by placing dried beetles in a two-part fast-set silicone (Pinkysil, Barnes, Australia) and then using these silicone moulds to create resin casts (Barnes, Australia). Casts were painted with primer, followed by blue and red acrylic paint, then a clear varnish.

We first identified suitable host trees, flowering *Eucalyptus leptopoda* mallee, with abundant active beetles (greater than 10 *T. bonvouloirii*). To ensure we could place beetle replicas in a similar position to active beetles and observe behaviour, we selected host trees that were less than 3 m tall. To set up the trial, we positioned one glossy and one matte replica beetle on the host tree using Blutack (Bostik, Victoria, Australia). Replica beetles were placed in similar positions (i.e. similar height, sun exposure and visibility) on outer leaves or branches. After the replica was placed, we observed each replica for 10 min, from at least 3 m away using binoculars. During this time, we recorded the duration and type of each interaction (mating attempt or walking on the model). Duration was recorded as an ordinal factor: 0 = extremely brief contact, 1 = less than 3 s, 2 = 3–5 s, 3 = 5–10 s, 4 = 10–20 s, 5 = more than 20 s. If a beetle did not fly out of sight after an interaction and then returned, we noted that it was a repeat interaction from the same individual. After 10 min, we swapped the glossy and matte replicas to account for possible effects of position, and conducted another 10-min observation period.

To investigate the effect of glossy reflections on beetle attraction, we ran three GLMMs in R. Before analysis, we removed any interactions that we documented as a repeat from the same beetle. The first model investigated whether replica beetle gloss influenced the total number of interactions. The model was fit with a Poisson error distribution and using the optimx optimization interface with the nlminb algorithm for improved convergence. We then assessed whether replica beetle gloss influenced the type of interaction (i.e. copulation or not) and the duration of interactions. For duration, we reclassified the response variable to short interactions (less than 10 s) and long interactions (greater than 10 s) because we had few observations classified as 2 (*n* = 1) or 3 (*n* = 4). Both variables (type of interaction and duration) were assessed with binomial GLMMs. All models included replica model gloss as the predictor variable, and site ID and position ID (i.e. location on the tree) as random effects. For the type of interaction model, position ID was removed from the final model to avoid singularity. Significance of fixed effects was assessed using Wald's chi-squared tests, and differences among groups were evaluated using means and 95% confidence intervals [19,20].

## 3. Results

Surface gloss enhanced long-distance attraction of male *T. bonvouloirii* beetles to interception traps, but only in sunny conditions ($\chi^{2}$ = 4.832, d.f. = 1, *p* = 0.0279; figure 2). Field-trapping occurred for 8 days, which included both sunny (*n* = 39 traps per treatment) and cloudy (*n* = 13 traps per treatment) periods. We captured 116 beetles during sunny conditions (gloss = 62, matte = 54) and 19 during cloudy conditions (gloss = 2, matte = 17). During sunny periods, glossy traps attracted jewel beetles 51% of the time, whereas matte traps attracted beetles only 14% of the time (maximum likelihood estimates from a generalized linear model; supplementary material, table S1). By contrast, during cloudy periods, the probability of attracting beetles did not differ between glossy and matte traps, and was similar to the matte trap probability in sunny conditions (glossy: 0.6%, matte: 6%; figure 2). These results align with predictions that surface gloss enhances visibility, but only under illumination conditions when the gloss is prominently visible.

Surface gloss also increased attraction of *T. bonvouloirii* to replica beetles at close range ($\chi^{2}$ = 9.153, d.f. = 1, *p* = 0.002; figure 3). During a 10-min observation period (*n* = 20 per treatment), glossy replica beetles attracted, on average, 1.3 *T. bonvouloirii* individuals per 10 min, whereas matte replica beetles attracted 0.6 beetles on average (supplementary material, table S1). This suggests that beetles use glossy reflections to help locate conspecifics on a host tree. Fifty-five per cent of the interactions involved the beetle attempting to mate with the replica, and 46% were longer than 10 s, independent of replica gloss (supplementary material, table S1).

## 4. Discussion

Our results show that jewel beetles are attracted to glossy cues, but only in sunny conditions. *T. bonvouloirii* beetles were attracted to gloss from both long and short distances, suggesting that glossiness can be used to locate host trees with conspecifics and to find conspecifics once near a host tree. Beetles are unlikely to have been attracted to our traps as a direct cue of water or food [10,22] because we have never observed beetles at water puddles at this site (on the few occasions when water was present), and *T. bonvouloirii* feeds on *Eucalyptus* flowers, which are matte. Conspecific attraction could indirectly signal a profitable host tree, but the strong male bias in our catches (99% male) suggests that beetles were more likely searching for mates than food. Using gloss to find conspecifics is likely relevant for other jewel beetle species. *Julodimorpha bakewelli* is famous for its attraction to beer bottles, a behaviour that is probably driven by the glossy reflections [23]. More broadly, many jewel beetle species are brilliantly coloured and glossy [7,24,25] and numerous such taxa are known to aggregate on host trees with other species. Surface gloss, however, is likely to be a reliable cue only under direct sunlight. Jewel beetles active during cloudy conditions are unlikely to use gloss for conspecific detection.

We did not detect any influence of glossiness on male copulation attempts or duration of the interaction with the replica beetle. This suggests that glossiness is not involved in assessment of potential mates by males. In principle, glossiness could contribute to species recognition or assessment of individual quality if it varies consistently among species or among individuals of the same species. However, the degree of surface reflection is strongly influenced by factors unrelated to species identity or individual quality, such as pollen or dust adhering to the cuticle or weather conditions, as we show here. Instead, jewel beetles may use chromatic properties during mate assessment, such as to distinguish conspecifics from heterospecifics [26]. It is possible that glossiness provides an effective long-distance cue of conspecifics, but becomes less informative at close range if bright surface reflections interfere with the assessment of chromatic cues, as was recently demonstrated in foraging bumblebees [10].

Several insect taxa can use reflections from glossy objects or water as a long-distance cue, including flies [12,27,28], bees [10], dragonflies [29], true bugs [22,30] and beetles [22,23,30]. This attraction has been most widely documented in aquatic insects and is generally understood to be driven by polarization cues used to detect water [22,31]. Glossy human-made structures or objects are also attractive to insects and can act as ecological traps. For example, glossy gravestones [29], solar panels [32], cars [30] and even beer bottles [23] can attract insects and may disrupt reproductive behaviours. These examples highlight the importance of gloss as a visual cue for insects, and suggest that our results have implications across a range of taxa.

Glossy reflections play a role in diverse visual behaviours, including predator avoidance [3,33], mate recognition [34], plant–pollinator signalling [10] and detection of water [31]. These functions have been observed across diverse animal groups, from insects to birds, suggesting that the visual role is widespread. Our results indicate that these functions of gloss are limited to objects in direct sunlight on sunny days. Other factors, however, may enhance the efficacy of gloss as a visual cue. Although our experiment used static stimuli, movement may further enhance detectability by creating high-contrast flashes of light [13,33]. Glossy objects are often not static. For example, beetles walk or fly between positions on the host plant, glossy flowers move in the wind and fiddler crabs wave their claws. The observer is also often moving, such as foraging or mate-searching animals looking for glossy targets. Flashes generally capture visual attention more effectively than static features [35]. Therefore, movement is likely to further enhance the salience of glossy cues across both long and short distances.

## Supplementary Material



## Data Availability

Data and analysis code are available on Dryad [36]. Supplementary material is available online [37].
