# Supplementary material for: Beetle gloss is a cue of conspecifics, but only in sunny conditions
Source: Biol Lett. 2026 Oct 21;22(10):20260358. doi: 10.1098/rsbl.2026.0358 (PMC13682924; doi:10.1098/rsbl.2026.0358)
Supplement: Supplementary file 1 [file rsbl.2026.0358.zip › rsbl.2026.0358/rsbl20260358_si_001.docx]

**Beetle gloss is a cue of conspecifics, but only in sunny conditions**

Amanda M. Franklin, Aika H. Young, Katrina J. Rankin, Aëto Apaix, Mark Hanlon, Avichal Tatu, Casper J. van der Kooi

**Supplemental Information**

**Methods**

*Reflectance measurements*

To quantify chromatic characteristics, we used a FLAME spectrometer and a PX-2 pulsed Xenon light source (Ocean Optics, USA) to measure reflectance of a 1 mm diameter patch from each sample. The light source was positioned at 20° from the sample normal and the collector probe at 0° from the normal. All measurements were taken relative to a 99% Spectralon white reflectance standard (Labsphere, USA) and we recorded three replicate measurements per sample. To identify paints and vinyl that best matched chromatic properties of real beetles, we visually compared spectra and used the pavo package in R [1-3] to quantify spectral shape by extracting the wavelength of peak reflectance and the maximum reflectance divided by minimum reflectance. With these measurements, we selected vinyl and paints that were most similar to real *Temognatha* beetles (Figure S1).

We also quantified the spread of reflected light across all angles, to fully characterize visual properties of our treatments and real beetles. To do this, we recorded reflectance spectra of beetles, artificial traps and beetle replicas as a function of angle of light incidence, using two rotatable optical fibres with a common rotation axis. One fibre illuminated the sample with a halogen-deuterium light source (Avantes AvaLight-DH-S-BAL) and the other fibre was connected to the spectrometer (Avantes StarLine Avaspec-ULS2048CL-EVORS). Illumination and observation angles ranged from -80 to 80° or -60 to 60° depending on the size of the sample, in 10° steps. The angular resolution of the setup has a Gaussian shape with a half-width of ~ 5° [4]. The spatial reflectance characteristics were compared by plotting the normalised reflectance as a function of illumination angle and observation angle [5] (Fig. 2, 3 main text).

**Figure S1: Reflectance of A) *Temognatha bonvouloirii*, B) glossy interception traps, C) matte interception traps, D) glossy replica beetles and E) matte replica beetles.** Peak reflectance (*λ_max_*) is at a similar wavelength for beetles, traps and replicas, and shape of reflectance spectra is similar for glossy and matte traps and for glossy and matte replica beetles.

*Interception traps*

Interception traps were created from blue plastic buckets (Big W, Australia) with corflute inserts. The inserts were covered in Oracal 551 marine blue PVC film (Orafol, NSW, Australia), which was the most similar colour match to the target *Temognatha* beetles. Both the buckets and the inserts were spray painted with clear glossy or matte varnish (Rust-oleum, 5 in 1 clear enamel; Vernon Hills, Illinois, USA). Each bucket had two inserts slotted together to create an X, and the top of one insert was bent to increase the range of directions that the gloss would be visible from.

*Beetle models*

We created replica models of *T. bonvouloirii* that varied only in surface gloss (either glossy or matte). Replicas were created from real beetles following our previously established protocol [8]. We created moulds of four *T. bonvouloirii* beetles by placing dried beetles (collected previously in Western Australia) in Pinkysil (Barnes, Australia). Beetles were removed and 10 casts of these beetles were created using polyurethane resin (Barnes, Australia). The casts were first painted with primer (grey surface primer, Tamiya, Irvine CA) followed by red and blue acrylic paint. The paints were colour matched to the real beetles, requiring a mixture of scarlet red (acrylic modelling colours, AK Interactive, Logroño, Spain), dark red (Model Colour range, Vallejo, Barcelona, Spain), blood red (acrylic modelling colours, AK Interactive, Logroño, Spain) and black (Game Colour range, Vallejo, Barcelona, Spain) to create the red (ratio: 8:8:4:1), and dark prussian blue (Model Colour range, Vallejo, Barcelona, Spain), royal blue (Model Colour range, Vallejo, Barcelona, Spain) and black (Game Colour range, Vallejo, Barcelona, Spain) to create the blue (ratio: 8:4:1). Half the replica beetle models were sprayed with high gloss varnish and the remaining replica beetles were sprayed with matte varnish (Mr Hobby, Mr Super Clear gloss or flat, Japan). This created glossy and matte models that differ only in surface gloss, not in colour or pattern.

*Experiment 1 data processing*

Prior to analysis, we removed unreliable collection periods. Two position pairs included one trap that was inadvertently positioned on a host tree. Traps were designed so that the glossy visual stimulus would be visible from a longer distance, rather than <1 meter away from above. Therefore, the treatments were likely not perceived as intended when placed on a host tree and these sampling periods were removed. In addition, to investigate the impact of weather on gloss attraction, we only included sampling periods that could easily be classified as sunny (i.e. no clouds or few clouds) or cloudy (i.e. overcast). There were several periods with patchy clouds that resulted in differing durations of sun exposure, which would add noise to the analysis. Final analyses were conducted with data from male *T. bonvouloirii* beetles because we captured very few female beetles (n = 2) or other species (n = 2).

**Supplemental Results**

*Experiment 1: Long-distance attraction*

Over eight days, we documented beetle attraction for 104 sampling periods. This included 78 periods in sunny conditions (i.e. 39 matte traps and 39 glossy traps) and 26 periods in cloudy conditions (i.e. 13 matte traps and 13 glossy traps). Male *T. bonvouloirii* were more likely to be captured in glossy traps, but only in sunny conditions (Table S1). In sunny conditions, 51% of glossy traps were likely to capture a beetle, compared to only 14% of matte traps. In cloudy conditions, both traps showed similar, low levels of capture success (glossy: 0.6%, matte: 6%; Figure 1). For the traps that captured beetles, trap gloss did not influence the number of beetles captured (*χ*^2^ = 2.188, df = 1, *p* = 0.139; Table S1). This may be because total catch is influenced more strongly by other factors, such as time of day, proximity to and position of the trap relative to the host tree, abundance of beetles in nearby host trees, or whether it was the initial versus subsequent sampling period.

*Experiment 2: Short-distance attraction*

Across all ten-minute observation periods (*n* = 20 for each treatment), we documented 64 interactions with our replica beetles. After removing interactions that were repeat visits from the same beetle, we recorded 54 interactions. Interactions were more likely to occur with glossy replica beetles compared to matte replica beetles (Table S1). In a ten-minute period, glossy replica beetles attracted 1.3 *T. bonvouloirii* on average whereas matte replica beetles attracted 0.6 beetles on average (Figure 1). Of these interactions, there was no impact of replica beetle gloss on whether the beetles attempted to copulate with the model, nor on whether the interaction was short (< 10s) or long (> 10s; Table S1).

**Table S1**: Statistical results from both experiments investigating whether gloss or weather conditions impacts buprestid long-distance attraction (Experiment 1) or short distance attraction (Experiment 2). Maximum likelihood estimates (MLEs) and 95% confidence intervals provide an indication of the effect size.

| **Response variable** | **Probability distribution** | **Explanatory variables** | **Chi-sq** | **d.f.** | **p-value** | **MLE (95% CI)** | | | |
| --- | --- | --- | --- | --- | --- | --- | --- | --- | --- |
| *Experiment 1* |  |  |  |  |  | Gloss, Sunny | Matte, Sunny | Gloss, Cloudy | Matte, Cloudy |
| Trap success (n = 104) | Binomial | Gloss | 3.699 | 1 | 0.0544 | 0.51 | 0.14 | 0.007 | 0.063 |
|  |  | Weather | 3.759 | 1 | 0.0525 | (0.22-79) | (0.034-0.42) | (0.0002-0.18) | (0.006-0.43) |
|  |  | **Gloss:Weather** | **4.832** | **1** | **0.0279** |  |  |  |  |
|  |  | **Sampling Duration** | **7.169** | **1** | **0.0074** |  |  |  |  |
|  |  |  |  |  |  | Gloss | | Matte | |
| Beetle count (n = 34) | Poisson | Gloss | 2.188 | 1 | 0.139 | 2.19 (1.35-1.69) | | 3.00 (1.69-5.32) | |
| *Experiment 2* |  |  |  |  |  | Gloss | | Matte | |
| Interaction count (n = 40) | Poisson | **Gloss** | **9.153** | **1** | **0.002** | 1.37 (0.64-2.89) | | 0.58 (0.25-1.32) | |
| Interaction type (n = 54) | Binomial | Gloss | 0.651 | 1 | 0.420 | 0.61 (0.40-0.79) | | 0.49 (0.24-0.74) | |
| Duration (n = 54) | Binomial | Gloss | 0.071 | 1 | 0.790 | 0.42 (0.26-0.61) | | 0.50 (0.25-0.75) | |

*Note: for experiment 2, interaction type and duration were quantified as binomial variables. For interaction type: 0 = no copulation attempt, 1 = copulation attempt; for duration 0 = short interaction (<10sec) and 1 = long interaction (>10sec). MLEs represent the probability of a copulation attempt or a long interaction, respectively.*

**References**

1.	R Core Team, *R: A Language and Environment for Statistical Computing*. 2024, R Foundation for Statistical Computing: Vienna, Austria.

2.	Maia, R., et al., *pavo: an R package for the analysis, visualization and organization of spectral data.* Methods in Ecology and Evolution, 2013. **4**(10): p. 906-913.

3.	Maia, R., et al., *pavo 2: new tools for the spectral and spatial analysis of colour in R.* Methods in Ecology and Evolution, 2019. **10**: p. 1097– 1107.

4.	Stavenga, D.G., M. Staal, and C.J. Van der Kooi, *Conical epidermal cells cause velvety colouration and enhanced patterning in Mandevilla flowers.* Faraday Discussions, 2020. **223**: p. 98-106.

5.	Dietz, A., J. Spaethe, and C.J. van der Kooi, *Dynamic visual effects enhance flower conspicuousness but compromise color perception.* Science Advances, 2025. **11**(48): p. eadz9010.

6.	Nakagawa, S. and I.C. Cuthill, *Effect size, confidence interval and statistical significance: a practical guide for biologists.* Biological reviews, 2007. **82**(4): p. 591-605.

7.	Halsey, L.G., *The reign of the p-value is over: what alternative analyses could we employ to fill the power vacuum?* Biology Letters, 2019. **15**(5): p. 20190174.

8.	Franklin, A.M., et al., *Cracks in the mirror hypothesis: high specularity does not reduce detection or predation risk.* Functional Ecology, 2022. **36**: p. 239-248.

**Data Availability**

Data and analysis code is available on Dryad (anonymous, private link for peer review):

<http://datadryad.org/share/LINK_NOT_FOR_PUBLICATION/VDb9oXAKcHZSrfLEcNq1UF97r9s-d2ARcGoGwTVjqos>
